# *AcWRKY22* activates *AcMYB210* to regulate light induced anthocyanin accumulation in kiwifruit leaves

**DOI:** 10.3389/fpls.2026.1944909

**Published:** 2026-10-05

**Authors:** Yan Zhang, Hao-lin Zheng, Shi-jiang Cui, Zhi-xia Huang, Xiao-fen Liu, Andrew C. Allan, Xue-ren Yin, Zhu Xie, Wen-qiu Wang

**Affiliations:** 1 School of Horticulture, Anhui Agricultural University, Hefei, Anhui, China; 2 Anhui Province Key Laboratory of Horticultural Crop Quality Biology, School of Horticulture, Anhui Agricultural University, Hefei, Anhui, China; 3 One Belt and One Road International Joint Research Center of Horticultural Products Quality and Post-Harvest Biotechnology in Anhui Province, School of Horticulture, Anhui Agricultural University, Hefei, Anhui, China; 4 New Zealand Institute for Bioeconomy Science, Auckland, New Zealand; 5 School of Biological Sciences, University of Auckland, Auckland, New Zealand

**Keywords:** AcMYB210, AcWRKY22, anthocyanin, kiwifruit, light, transcriptional regulation

## Abstract

**Background:**

Plant leaves often develop red coloration in response to aging and environmental changes. High light or continuous light treatments can elevate anthocyanins, which we observed in normally green kiwifruit leaves.

**Methods and results:**

The underlying mechanism in this response was investigated using transcriptomic analysis. Results revealed that multiple anthocyanin biosynthesis genes, particularly Flavonoid-3-O-glucosyltransferase (*AcF3GT1a*), (*AcF3GT4*) can convert unstable anthocyanidins into stable colored anthocyanins, and glutathione-S-transferase (*AcGST1*) is responsible for the safe delivery of anthocyanins from the endoplasmic reticulum to the vacuole, were significantly upregulated under continuous light light-stress treatments. *AcMYB210*, a member of the S6 MYB subfamily in kiwifruit with previously unknown function, was specifically induced. Dual-luciferase, yeast one-hybrid, and homologous genetic transformation assays confirmed that *AcMYB210* directly binds to and activates the promoters of anthocyanin pathway genes, thereby promoting anthocyanin accumulation in kiwifruit leaves. A light-induced transcription factor, *AcWRKY22*, was found to directly bind and activate the *AcMYB210* promoter. In addition, *AcMYB210* was highly expressed in plants whose leaves turned red after waterlogging stress.

**Conclusions:**

In summary, our data reveal the critical role of the *AcWRKY22-AcMYB210* module in mediating light signal regulation of anthocyanin biosynthesis in kiwifruit leaves, providing new insights into the regulation of kiwifruit anthocyanin biosynthesis by light signals.

## 1 Introduction

Anthocyanins are water-soluble flavonoid pigments widely distributed in vascular plants, conferring red, purple, and blue coloration to various tissues, where they perform multifaceted physiological functions, including photoprotection against excess high light, reactive oxygen species (ROS) scavenging, UV shielding, pollinator attraction, and stress signal transduction (Liu et al., 2018; Zheng et al., 2020; Cappellini et al., 2021; Naing and Kim, 2021). The accumulation of anthocyanins is influenced by various factors, including genotype-specific control, developmental stage, and environmental conditions (Kuang et al., 2025). Anthocyanins often accumulate in vegetative tissues under stress conditions, serving as an indicator of plant health status.

Kiwifruit (*Actinidia* spp.) is an economically important crop globally, renowned for its high vitamin C and flavonoid content. However, most existing research on kiwifruit anthocyanin biosynthesis has focused on pigmentation in the fruit flesh and skin (Liu et al., 2023; Yu et al., 2024; Song et al., 2025). Red pigment accumulation is frequently observed in kiwifruit leaves, yet the conditions leading to its formation and the underlying molecular mechanisms remain poorly understood.

Light is universally recognized as one of the most influential environmental factors controlling anthocyanin biosynthesis (Li et al., 2019; Ma et al., 2021), including photoperiod, light intensity, and light quality. For example, high light can induce reddening in *Arabidopsis* and other plants (Ma et al., 2021; Xing et al., 2024) while ultraviolet (UV) and blue light have also been reported to promote anthocyanin accumulation in plants as diverse as apple and lettuce (Fang et al., 2019a; Zhang et al., 2026). Plants perceive light signals through photoreceptors, including phytochromes (red/far-red light receptors, PhytochromeA-E: PHYA-E), cryptochromes and phototropins (blue/UV-A receptors, Cryptochrome1-2: CRY1–2 and Phototropin: PHOT1-2), and the UV-B receptor UV Resistance locus8 (UVR8) (Chen and Chory, 2011; Yin and Ulm, 2017). In the light-regulated pathway, the ELONGATED HYPOCOTYL 5 (HY5) transcription factor acts as a central integrator, directly binding to G-box or ACE elements in the promoters of anthocyanin biosynthetic genes and activating their expression (Bursch et al., 2020; Zhang et al., 2026). Conversely, the CONSTITUTIVELY PHOTOMORPHOGENIC 1 (COP1) E3 ubiquitin ligase targets HY5 for degradation in darkness, thereby repressing photomorphogenic development and anthocyanin accumulation (Chen and Chory, 2011). The interplay between photoreceptors, COP1, and HY5 establishes a delicate light-dependent switch that determines the extent of anthocyanin accumulation.

Downstream of these core signaling components, the transcriptional activation of anthocyanin structural genes is primarily mediated by the MYB-bHLH-WD40 (MBW) complex, composed of R2R3-MYB, basic helix-loop-helix (bHLH), and WD40 repeat proteins (Xu et al., 2015; Liu et al., 2021; Jiang et al., 2024; Li et al., 2024). Among these, R2R3-MYB transcription factors of the sub-group clade 6 act as key specificity determinants (Rodrigues et al., 2021). In *Arabidopsis*, *AtPAP1* and *AtPAP2* (Production of Anthocyanin Pigment) are well-established activators that respond to high light and induce the expression of anthocyanin biosynthetic genes such as chalcone synthase (*CHS*), chalcone isomerase (*CHI*), flavanone 3-hydroxylase (*F3H*), dihydroflavonol 4-reductase (*DFR*), anthocyanidin synthase (*ANS*), and flavonoid-3-*O*-glucosyltransferase (*UFGT*) (Zhou et al., 2012; Lee et al., 2024; Xing et al., 2024). Similarly, in apple, *MdMYB1* and *MdMYB10* confer light-responsive red pigmentation in fruit skin and flesh, respectively (Hu et al., 2016). In pear, *PyMYB10* and *PyMYB114* have been shown to regulate anthocyanin accumulation under different light qualities (Bai et al., 2019; Alabd et al., 2022). The conservation of this regulatory module across diverse species underscores its evolutionary significance.

In kiwifruit, several MYB transcription factors involved in fruit anthocyanin regulation have been characterized, with most studies focusing on fruit flesh color. There are at least three S6 MYB members in kiwifruit, including *MYB10*, *MYB110* and *MYB210* (Rodrigues et al., 2021). *MYB10* and *MYB110* are associated with red inner pericarp and whole flesh kiwifruit (Wang et al., 2022; Li et al., 2026),. In addition *AcMYB123* has been shown to be involved in the anthocyanin accumulation in the inner pericarp of kiwifruit and shares closest sequence homology to AtMYB123 (Wang et al., 2019).

In this study, we found that continuous light treatment induced anthocyanin accumulation in kiwifruit leaves. Transcriptome profiling identified *AcF3GT1a*, *AcF3GT4*, and *AcGST1* as candidate key genes involved in light-induced anthocyanin biosynthesis. We also found that *AcMYB210*, a distinct member of the kiwifruit S6 MYB subfamily with previously unknown function, was induced by light treatment. AcMYB210 positively regulated the promoters of anthocyanin biosynthesis genes and was elevated by the light-induced transcription factor *AcWRKY22.* Additionally, *AcMYB210* was also upregulated under other abiotic stresses, so may serve as a marker gene for anthocyanin accumulation in kiwifruit in response to stress.

## 2 Materials and methods

### 2.1 Plant materials and treatment

The kiwifruit cultivar *Actinidia chinensis* cv. ‘Donghong’ was used in this study. ‘Donghong’ seedlings of control group were grown in a plant incubator with 16:8 white light: dark photoperiod (The light intensity is 8000 lux), and the treated group were cultured under 24-hour white light for two weeks. After two weeks, photographs of the leaves were taken to record the changes in anthocyanin phenotypes. The leaves were then rapidly frozen in liquid nitrogen and stored at -80 °C refrigerator for subsequent RNA extraction and transcriptome sequencing.

For waterlogging treatment, using kiwifruit plants with a plant height of approximately 30 cm as materials. The control group was watered normally to maintain substrate moisture. The treatment group was submerged in water for three days and then transferred back to normal conditions to recover. Plants were grown in a climate chamber at 25 °C. After 15 days of recover, some leaves of the treatment group showed obvious anthocyanin accumulation. Leaves from both the control and treatment groups were collected and immediately frozen in liquid nitrogen for subsequent extraction of anthocyanins and RNA.

### 2.2 RNA extraction and qRT-PCR reactions

Extracted RNA from kiwifruit and *Nicotiana benthamiana* leaves according to the previously described method (Wang et al., 2022). First-strand cDNA was synthesized from 1 μg of total RNA using a PrimeScript™ RT Reagent Kit with gDNA Eraser (Vazyme, China) following the manufacturer’s protocol. qRT-PCR was performed on the iCycler iQ5 real-time PCR detection system (Bio-Rad, USA) using PerfectStart Green qPCR SuperMix (TransGen Biotech, Chian). Each 20-μL reaction contained 10 μL of 2× SYBR Green Master Mix, 0.4 μL each of forward and reverse primers (10 μM), 2 μL of diluted cDNA, and 7.2 μL of RNase-free water. The thermal cycling program was as follows: 95 °C for 30 s, followed by 40 cycles of 95 °C for 5 s and 60 °C for 30 s. Melting curve analysis was performed from 65 °C to 95 °C to confirm amplification specificity. Relative expression levels were calculated using the 2^^−ΔΔCt^ method. All reactions were performed in three biological replicates and three technical replicates. Primer sequences used in this study are listed in **Supplementary Table S1**.

### 2.3 Transcriptome sequencing and analysis

RNA extracted from the kiwifruit leaves (control and light treatment) with three replicates were used for transcriptome sequencing and analysis. The RNA-seq was performed on the Illumina sequencing platform by Personalbio (Nanjin, China). After removing unqualified reads (adapters, poly-N and low-quality), clean reads were obtained and the Red5 genome database was use as reference genome (Pilkington et al., 2018). Gene expression levels were estimated using fragments per kilobase of transcript per million fragments mapped (FPKM). The differential expression analysis of genes was performed using DESeq2. By default, the criteria for screening differentially expressed genes are: absolute log2 fold change (|log2FoldChange|) > 1, and significance P-value < 0.01. The statistical enrichment of DEGs in the Kyoto Encyclopedia of Genes and Genomes (KEGG) pathway was analyzed using KOBAS software (Kanehisa et al., 2016). The Gene Ontology (GO) enrichment analysis of DEGs were conducted with the GOseq R package (Mi et al., 2019).

### 2.4 Extraction and calculation of anthocyanin content

Frozen samples of kiwifruit and *Nicotiana tabacum* leaves were powdered. Weighed (0.05 g) powder, was added to 600 μL of 1% hydrochloric acid-methanol solution, and then store at 4 °C in the dark for 2 hours. After 400 μL of ddH_2_O was added, mixed gently, and then mixed with 1 mL of chloroform. The mixture was centrifuged (6,000 rpm, 5 minutes) and the supernatant collected. 800 μL of the supernatant was diluted to 1.6 mL with 60% 1% hydrochloric acid-methanol solution (prepared by mixing 20 mL ddH_2_O with 30 mL 1% hydrochloric acid-methanol). The absorbance was measured at 530 nm and 657 nm using a spectrophotometer. The anthocyanin content is calculated by the calculation ((A530-A657) ×fold/mg FW tissue) ×1000.

### 2.5 Yeast two-hybrid assay

The coding sequences (CDS) of *AcMYB210* and *AcbHLH5* were cloned into the bait vector pGBKT7 and prey vector pGADT7, respectively. The resulting recombinant plasmids pGBKT7-AcMYB210 and pGADT7-AcbHLH5 were verified by DNA sequencing. For co-transformation, 100 µL of AH109 competent cells were mixed with pGBKT7-AcMYB210 and pGADT7-AcbHLH5 plasmid, 10 µL of denatured carrier DNA (10 mg/mL), and 500 µL of PEG/LiAc solution. The mixture was vortexed thoroughly and incubated at 30 °C with shaking at 220 rpm for 30 min. The mixture was gently inverted to mix, followed by heat shock at 42 °C for 40 min (with gentle mixing every 10 min). The tube was chilled on ice for 2 min, and 100 µL of the transformation mixture was spread onto selective SD/-Leu/-Trp agar plates. Plates were incubated at 30 °C for 3–4 days until colonies appeared. Subsequently, the interaction was verified on the SD/-Leu-Trp-His-Ade plate. Primer sequences used in this assay are listed in **Supplementary Table S1**.

### 2.6 LCI assay

The full-length CDS of *AcMYB210* and *AcbHLH5* were individually cloned into pCAMBIA1300-nLUC (nLUC) and pCAMBIA1300-cLUC (cLUC) vectors, respectively. These recombinant constructions of AcMYB210-nLUC and AcbHLH5-nLUC were transformed into *Agrobacterium tumefaciens* GV3101 cells. *Nicotiana benthamiana* leaves were infiltrated with an Agrobacterium mixture (OD_600_ = 1) carrying the recombinant plasmids. Two days after infiltration, luciferase signaling was detected by a Tanon-5200 plant visualization device (Shanghai, China). The relevant primers utilized are provided in **Supplementary Table S1**.

### 2.7 Yeast one-hybrid assay

The CDS of the *AcMYB210* and *AcWRKY22* were cloned into the pGADT7 vector, and the promoter sequence of *AcMYB210*, *AcF3GT1a* and *AcGST1* were cloned into the pAbAi vector. The pABAi-AcMYB210, pABAi-AcF3GT1a and pABAi-AcGST1 plasmid was linearized by digestion with BstBI or BbsI restriction enzyme (NEB) at 37 °C for 1 h, followed by enzyme inactivation. The linearized plasmids were transformed into Y1H-gold competent cells and spread on SD/-Ura plates. The AD-AcMYB210 plasmid was transferred into the Y1H-gold cells containing pABAi-AcF3GT1a and pABAi-AcGST1, and the AD-AcWRKY22 plasmid was transferred into the Y1Hgold competent cells containing pABAi-AcMYB210. To assess interaction, the fused yeast strains were selected on SD/-Leu with the antibiotic AbA. Primer sequences used in this study are listed in **Supplementary Table S1**.

### 2.8 Dual-luciferase reporter assays

The promoter fragments (approximately 2000 bp upstream of ATG start site) of *AcF3GT1a*, *AcGST1*, and *AcMYB210* were cloned and inserted into the pGreenII 0800-LUC (LUC) plasmid as reporter vector, these recombinant constructs were introduced into *A. tumefaciens* GV3101. The CDS of *AcMYB210* and selected transcription factors were cloned and inserted into the pSAK277 and these recombinant constructs were introduced into *A. tumefaciens* strain EHA105. Next, a mixed *Agrobacterium* suspension (OD_600_ = 0.75) harboring the recombinant plasmids was transiently injected into the six leaves of *N. benthamiana*. These *N. benthamiana* plants were then grown in artificial climate chamber. Three days after infection, Firefly luciferase (LUC) and Renilla luciferase (REN) activities were measured using Synergy H1 (Biotek, USA) with the Dual Luciferase Reporter Gene Assay Kit (YEASEN, Shanghai, China).

### 2.9 Transcriptional activation assays of *AcMYB210* in *N. benthamiana*

The CDS sequence of *AcMYB210* was fused with the GAL4 binding domain (GAL4 DBD) as effectors. The pGAL4DBD and pGAL4DBD-VP16 were used as the negative and positive effectors, respectively. pGAL4-LUC, in which a minimal 35S promoter plus five copies of GAL4 binding elements drive the firefly luciferase CDS, was used as the reporter. Firefly luciferase (LUC) and Renilla luciferase (REN) activities were measured using Synergy H1 (Biotek, USA) with the Dual Luciferase Reporter Gene Assay Kit (YEASEN, Shanghai, China).

### 2.10 Subcellular localization of AcMYB210

The CDS of *AcMYB210* was inserted into the pBin-35S-GFP vector to generate the recombinant vector 35S:AcMYB210-GFP. The recombinant vector was transformed into *A. tumefaciens* GV3101. Four-week-old *N. benthamiana* seedlings grown under a 16 h light/8 h dark photoperiod were used for transient infiltration. The bacterial suspension was gently injected into *N. benthamiana* leaves using a 1 mL syringe without a needle. Infiltrated *N. benthamiana* plants were maintained under normal growth conditions for 48–72 h to allow sufficient protein expression. The GFP and mCherry fluorescence of *N. benthamiana* leaves were detected using confocal laser scanning microscopy (A1, Nikon, Tokyo, Japan). The empty GFP vector used as a control, and the red fluorescent protein mCherry-NLS was co-expressed as a nuclear marker. Primers used for this assay are listed in **Supplementary Table S1**.

### 2.11 Transient overexpression assay in *N. tabacum*

The full-length sequences of *AcMYB210* and *AcbHLH5* were inserted into pSAK277 vector to generate the pSAK277-AcMYB210 and pSAK277-AcbHLH5. These recombinant constructs were introduced into *A. tumefaciens* strain EHA105. Cultured *A. tumefaciens* strains were resuspended in infiltration buffer (10 mM MES, pH 5.6, 10 mM MgCl_2_, and 150 μM acetosyringone) to a final OD_600_ of 1.0. The resulting suspensions carrying AcMYB210+AcbHLH5, empty vector (EV)+AcMYB210, EV +AcbHLH5 and EV were infiltrated into the *N. tabacum* leaves. Following infiltration, the plants were maintained in a greenhouse under a 16-h-light/8-h-dark photoperiod for 6 days. The infiltrated leaves were photographed at 6 days after infiltration, after which the infiltrated regions were sampled for subsequent analyses. Each experiment was conducted with three independent biological replicates. All primers used in this experiment are listed in **Supplementary Table S1**.

### 2.12 Stable transformation of *AcMYB210* in kiwifruit

The CDS of *AcMYB210* was transformed into the pSAK277 vector and introduced into *A. tumefaciens* strain EHA105. Transformation was performed on young leaves excised from tissue-cultured ‘Donghong’ seedlings, following the procedure detailed in our previous study (Wang et al., 2022). Putative transformants were selected on medium supplemented with kanamycin (50 mg L^-^¹), and confirmed positive lines were validated at the RNA level, *via* qRT-PCR. Primer sequences used for vector construction and transgenic identification are listed in **Supplementary Table S1**.

## 3 Results

### 3.1 Continuous white light treatment induces anthocyanin accumulation in kiwifruit leaves

Nighttime supplemental lighting is used in in horticulture crops to promote fruit color change or improve quality. but the effects of continuous light exposure on tree physiology remain unclear. Therefore, we treated kiwifruit plants with continuous 24-hour white light. After two weeks of treatment, we found some leaves turn red, anthocyanin accumulation was observed at the leaf margins, whereas leaves under a normal photoperiod remained green (**Figure 1A**). Quantitative analysis further confirmed that anthocyanin content in the treated plants was significantly higher than that in the control group. These results demonstrate that photoperiod is a factor affecting anthocyanin accumulation in kiwifruit.

**Figure 1:**
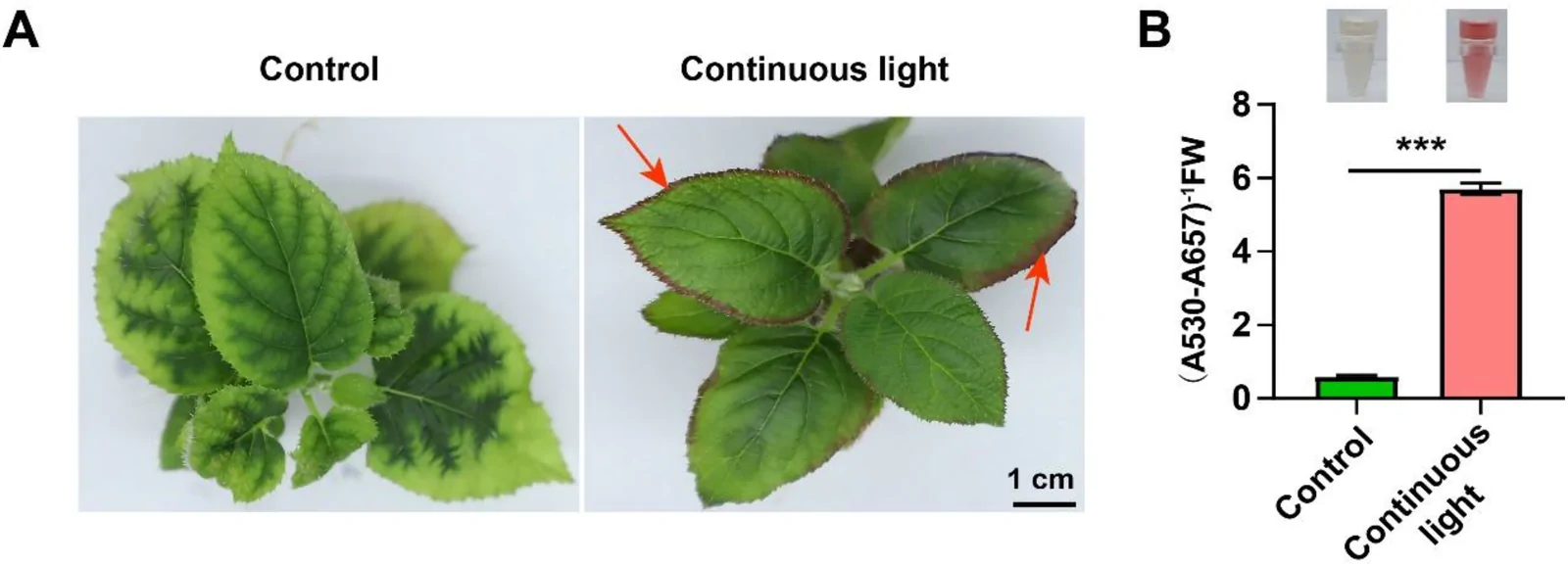
Continuous white light treatment promotes anthocyanin biosynthesis in kiwifruit leaves. **(A)** Photographs of kiwifruit leaves under control and continuous light treatment. Scale bar=1 cm. **(B)** Anthocyanin content in control and continuous light-treated leaves. Two-tailed Student’s *t*-test was conducted for statistical analysis and significant differences are marked with asterisks (***P < 0.001).

### 3.2 Downstream anthocyanin biosynthetic genes *AcF3GT1a*, *AcF3GT4*, and *AcGST1* respond to continuous light treatment

Transcriptome sequencing was performed on leaves subjected to normal and continuous light treatments. The Q20 value of the tested samples ranged from 99.35% to 99.41%, and the Q30 value ranged from 98.358% to 98.52%, indicating the high quality of the reads. The *Actinidia chinensis* Red5 genome was used as the reference genome; the average mapping rate reached 86.52%, and the average GC content was 45.98% (**Supplementary Table S2**). Principal component analysis (PCA) was employed to evaluate the similarities and differences between the samples. The results demonstrated a clear separation between the samples subjected to normal light conditions and those under continuous light treatment (**Figure 2A**). The correlation of gene expression levels between samples serves as a critical indicator for assessing experimental reliability and the appropriateness of sample selection. The results demonstrate that the correlation coefficients among between replicates subjected to different treatments approximates 1 (**Figure 2B**), indicating a high degree of similarity in their expression profiles. Further differential analysis of gene expression identified a total of 3,660 differentially expressed genes (DEGs), among which 2,288 were up-regulated and 1,372 were down-regulated (**Supplementary Figure S1**). Cluster analysis of DEGs revealed that their expression patterns varied significantly between treatment and control (**Supplementary Figure S2**). The GO and KEGG enrichment analyses were performed to determine the principal biological functions of the differentially expressed genes. The top five in the GO enrichment are: response to stimulus, response to chemical, DNA-binding transcription factor activity, response to oxygen-containing compound, and regulation of hormone levels (**Figure 2C**; **Supplementary Table S3**). The top five pathways in the KEGG enrichment analysis are Phenylpropanoid biosynthesis, Plant hormone signal transduction, Isoflavonoid biosynthesis, Flavonoid biosynthesis, and ABC transporters, all of which are associated with anthocyanin metabolism (**Figure 2D**; **Supplementary Table S4**).

**Figure 2:**
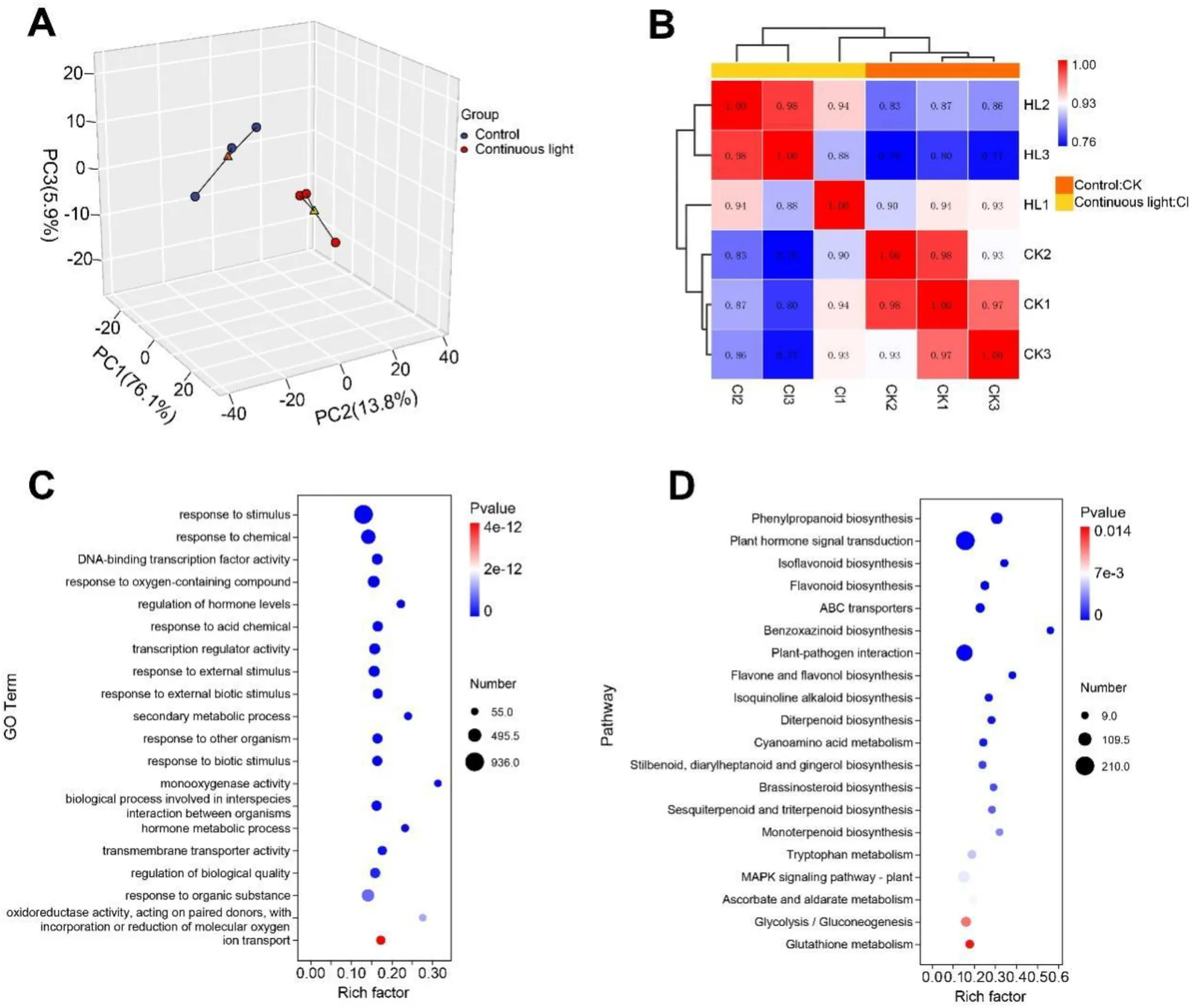
Analysis of RNA sequencing results. **(A)** PCA of transcriptome data from control and continuous-light treated samples. Each dot represents one biological replicate. Blue dots correspond to the control group, and red dots represent the continuous-light treatment group. **(B)** Pearson correlation coefficient heatmap among all biological replicates. Color gradient from blue to red indicates the correlation coefficient ranging from 0.76 to 1.00. CK1-CK3 represent biological replicates of the control group; HL1-HL3 represent biological replicates under continuous-light treatment. Hierarchical clustering was performed based on gene expression profiles. **(C)** GO enrichment analysis of differentially expressed genes (DEGs), bubble color indicates the significance of enrichment (*P*-value). **(D)** KEGG pathway enrichment analysis of differentially expressed genes, bubble color indicates the significance of enrichment (*P*-value).

In our previous study (Li et al., 2026), genes involved in anthocyanin metabolism in kiwifruit have been characterized. We analyzed the expression profiles of these anthocyanin metabolism genes and found that most anthocyanin genes (*AcCHS2*, *AcCHS3*, *AcCHS4*, *AcCHI1*, *AcF3H1*, *AcF3H2*, *AcF3’H2*, *AcF3’H3*, *AcF3’H4*, *AcDFR1*, *AcDFR2*, *AcANS1*, *AcANS4*, *AcF3GT1a*, *AcF3GT4*, and *AcGST1*) were up-regulated after continuous light treatment. Among these, *AcF3GT1a*, *AcF3GT4*, and *AcGST1* showed almost no expression in controls, rising to very high levels under continuous light treatment (**Figure 3**; **Supplementary Table S5**), suggesting that *AcF3GT1a*, *AcF3GT4*, and *AcGST1* play more critical roles in the accumulation of anthocyanins in kiwifruit leaves in response to light. These results are similar to our previous findings (Li et al., 2026), indicating that *F3GT* and *GST* members are key metabolic genes limiting anthocyanin synthesis in kiwifruit.

**Figure 3:**
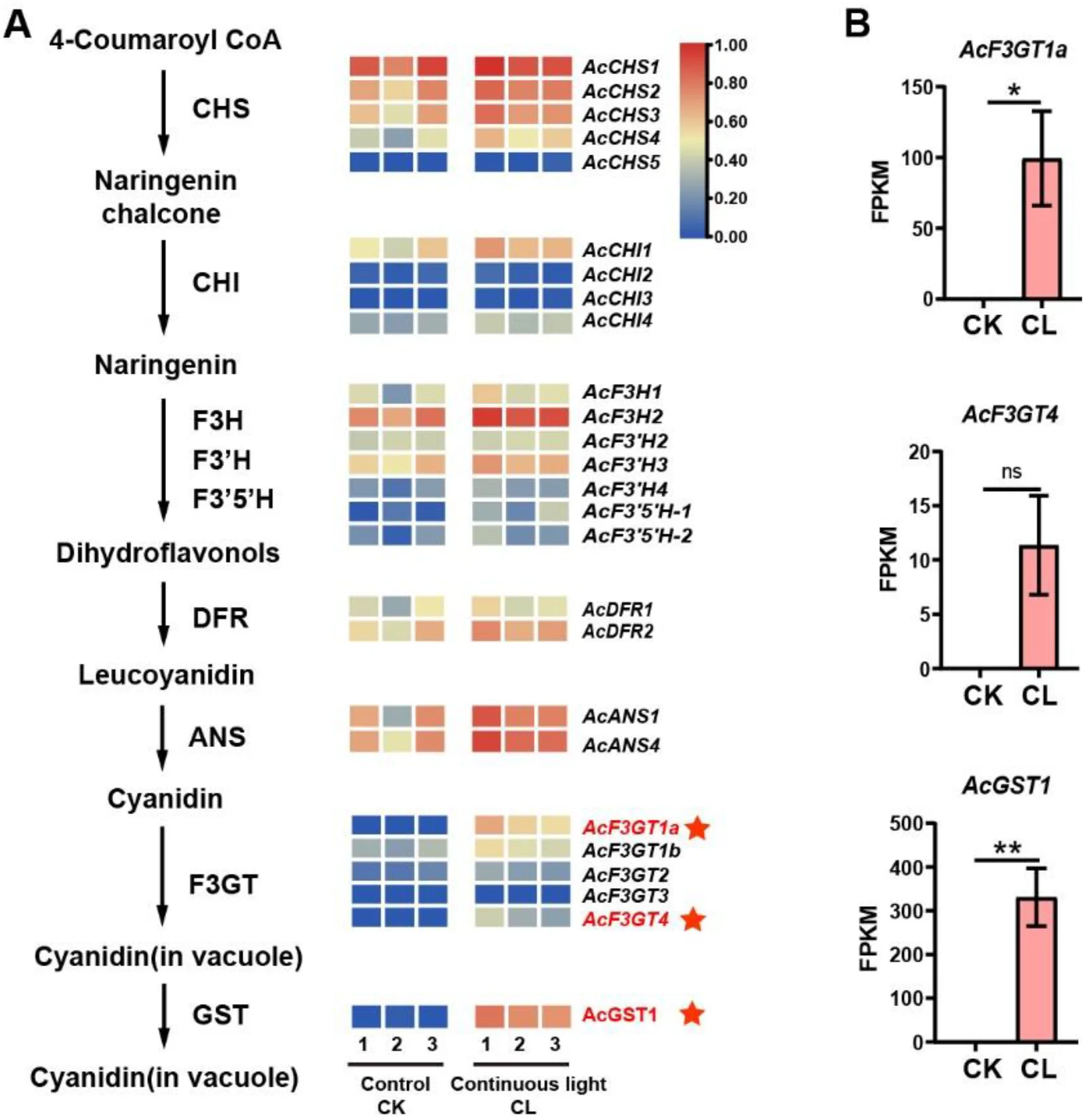
Expression patterns of genes involved in anthocyanin synthesis. **(A)** Heatmap showing expression of key genes in the anthocyanin synthesis pathway in leaves of ‘Donghong’ under continuous light treatment. **(B)** Expression characteristics of *AcF3GT1a*, *AcF3GT4*, and *AcGST1* expression. Two-tailed Student’s *t*-test was conducted for statistical analysis and significant differences are marked with asterisks (*P < 0.05, **P < 0.01).

### 3.3 A S6 R2R3 MYB member *AcMYB210* is up-regulated under continuous light treatment

Previous studies have reported that kiwifruit *AcMYB10*, *AcMYB110*, and *AcMYB210* belong to the S6 MYB subfamily (Rodrigues et al., 2021), the same clade as *AtPAP1*, *AtMYB113*, and *AtMYB114* which regulate anthocyanin biosynthesis in *Arabidopsis* (Rodrigues et al., 2021). Interestingly, the previously identified two genes, *AcMYB10* and *AcMYB110*, which control flesh color, were not expressed in either control or treated plants. In contrast, *AcMYB210*, whose function was previously unknown, was significantly up-regulated under continuous light treatment, suggesting that *AcMYB210* may be a key transcription factor regulating foliar anthocyanin biosynthesis (**Figure 4A**).

**Figure 4:**
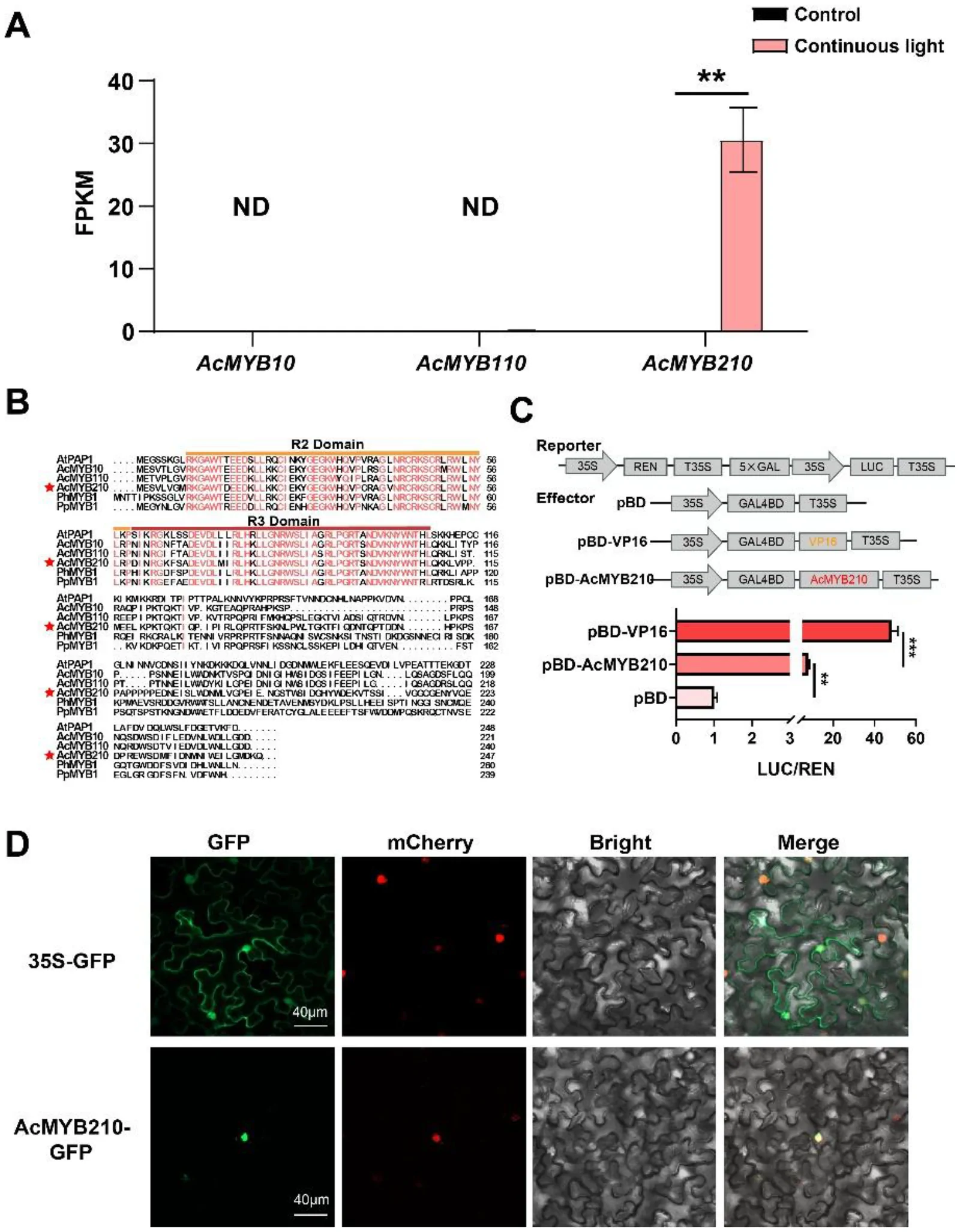
Expression pattern and functional characteristics of AcMYB210. **(A)** Expression patterns of *AcMYB10*, *AcMYB110* and *AcMYB210* from RNA-seq. **(B)** Alignment of the AcMYB210 amino acid sequence with those of other anthocyanin-related MYB transcription factors from other plants. **(C)** Transcriptional activity assay of *AcMYB210*. **(D)** Subcellular localization of *AcMYB210* in *N. benthamiana* leaves. The AcMYB210-GFP fusion protein was localized exclusively to the nucleus. Two-tailed Student’s *t*-test was conducted for statistical analysis and significant differences are marked with asterisks (**P < 0.01, ***P < 0.001).

Protein sequence alignment was performed to clarify the structural characteristics of AcMYB210, revealing that AcMYB210 shares structural similarity with AtPAP1, AcMYB10, AcMYB110, PhMYB1, and PpMYB1, and contains a typical R2R3 domain (**Figure 4B**). To evaluate the transcriptional activity of AcMYB210 *in vivo*, reporter vectors containing 5×GAL4 activation domains upstream of LUC were transiently co-expressed with effector vectors (pBD, pBD-AcMYB210, and pBD-VP16) in *N. benthamiana* leaves. Similar to the positive control pBD-VP16, co-expression of the reporter vector with pBD-AcMYB210 significantly increased the LUC/REN ratio compared with the negative control pBD (**Figure 4C**). To determine the subcellular localization of AcMYB210, the 35S:AcMYB210-GFP fusion protein was transiently expressed in the leaves of six-week-old *N. benthamiana*. The 35S: GFP control was detected in both the cytoplasm and the nucleus (**Figure 4D**), whereas the 35S:AcMYB210-GFP fusion protein was observed exclusively in the nucleus, indicating that AcMYB210 is a nuclear-localized protein. Together, these data suggest that *AcMYB210* functions as a transcriptional activator.

### 3.4 *AcMYB210* positively regulates expression of key anthocyanin biosynthesis genes

The promoters of the *AcF3GT1a* and *AcGST1* were cloned and constructed into the dual-luciferase reporter vector (**Figure 5A**). Dual-luciferase assays showed that *AcMYB210* could strongly activate the promoters of *AcF3GT1a* and *AcGST1* (**Figure 5B**). Y1H assay was performed to further confirm that AcMYB210 can bind to the promoters of *AcF3GT1a* and *AcGST1* (**Figure 5C**). These results indicate that *AcMYB210* directly binds and activates the key anthocyanin biosynthesis genes *AcF3GT1a* and *AcGST1*.

**Figure 5:**
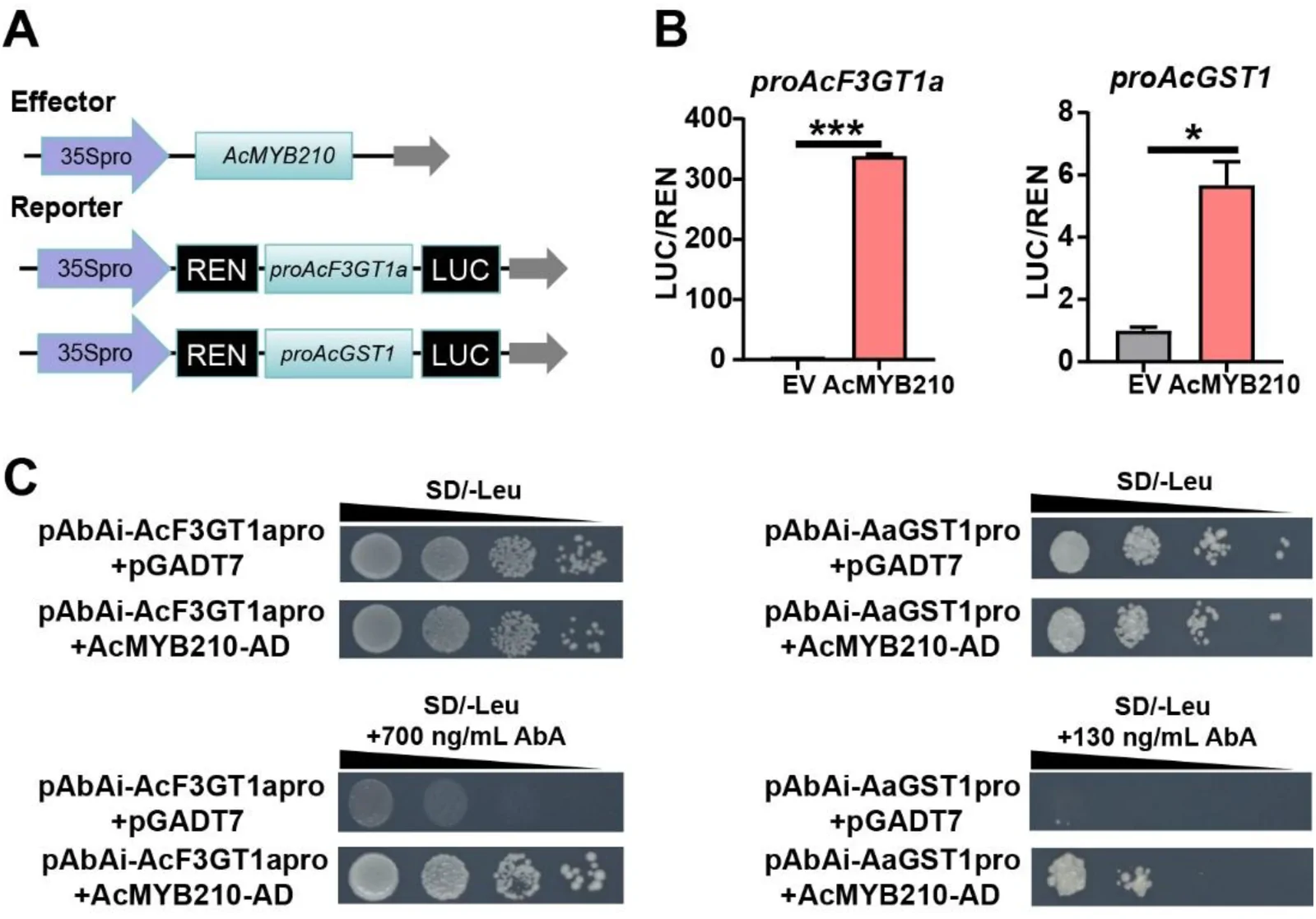
*AcMYB210* positively regulates *AcF3GT1a* and *AcGST1*. **(A)** Schematic diagram of the dual luciferase system. **(B)** Regulatory effect of *AcMYB210* on the promoters of *AcF3GT1a*, and *AcGST1*. **(C)** Y1H assays showing the interaction between AcMYB210 and the promoters of *AcF3GT1a* and *AcGST1.* The AD vector served as the negative control. Two-tailed Student’s *t*-test was conducted for statistical analysis and significant differences are marked with asterisks (*P < 0.05, ***P < 0.001).

### 3.5 *AcMYB210* promotes anthocyanin biosynthesis both in *N. tabacum* and kiwifruit

Transient *AcMYB210* expression in *N. tabacum* leaves detected no anthocyanin accumulation. However, when we co-expression AcMYB210 with partner protein AcbHLH5 (**Supplementary Figure S3**), which is homologous gene of Arabidopsis TT8 (Liu et al., 2021), produced strong activation. After five days, no pigmentation was detected in leaves infiltrated with the empty vector (EV), AcMYB210+EV and AcbHLH5+EV. In contrast, obvious pigmentation was found at the infiltration region transformed with AcMYB210+AcbHLH5 (**Figure 6A**). The visual phenotypes were fully consistent with both the measured anthocyanin content and the expression profiles of anthocyanin-related genes, mainly include *NtCHS*, *NtCHI*, *NtF3H*, *NtF3’H*, *NtDFR*, *NtANS*, and *NtUFGT* (**Figures 6B, C**).

**Figure 6:**
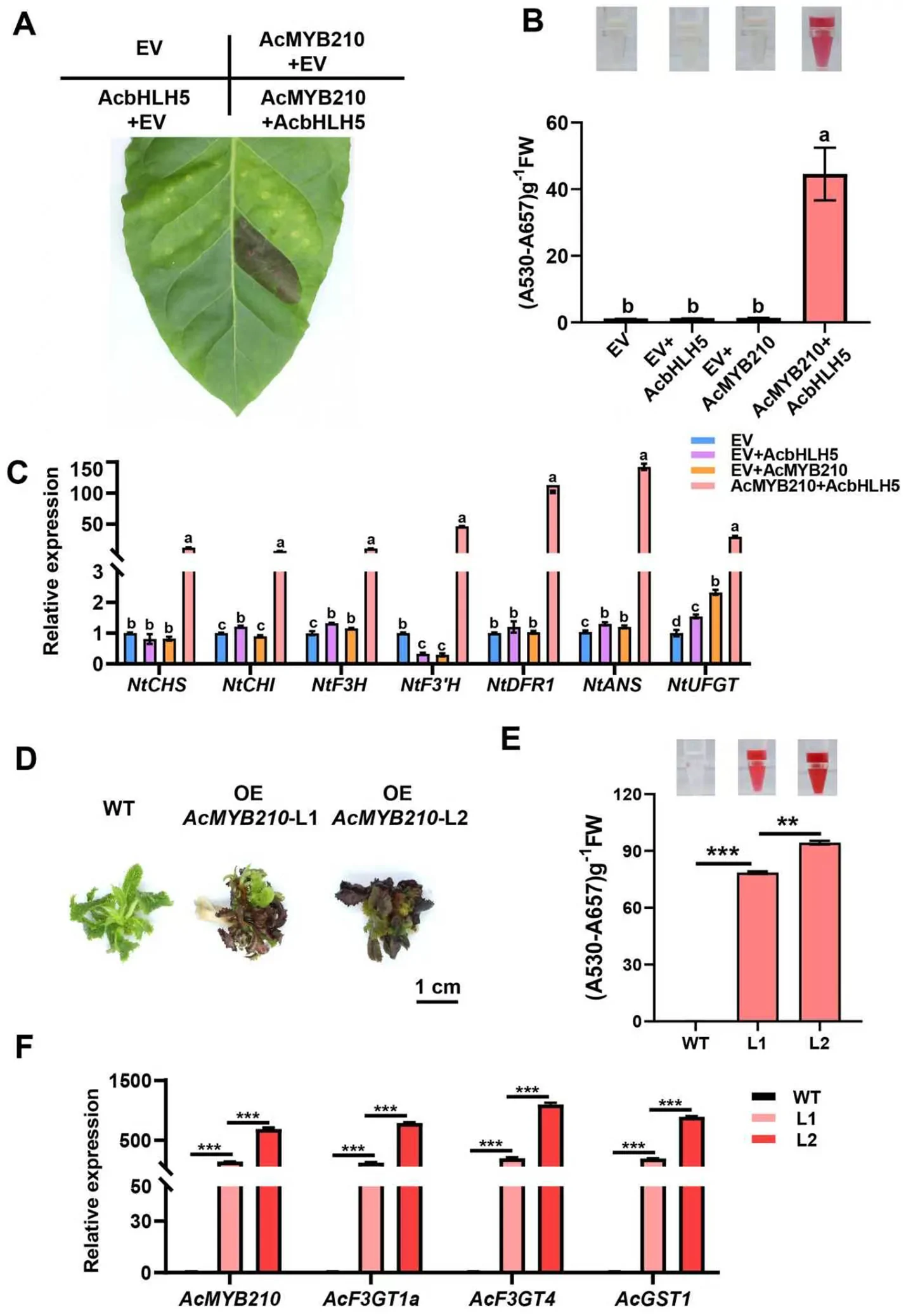
*AcMYB210* promotes anthocyanin synthesis both in *N. tabacum* and kiwifruit. **(A)** Ectopic transiently co-expression of *AcMYB210* and *AcbHLH5* leads to the anthocyanin accumulation in *N. tabacum* leaves. **(B)** Total anthocyanin content in different transient groups. **(C)** Expression levels of anthocyanin biosynthesis genes in different transient groups. Data are presented as mean ± SD. Different lowercase letters above the bars indicate significant differences among groups (one-way ANOVA followed by Tukey’s HSD *post-hoc* test, *p* < 0.05). **(D)** The phenotype of stably overexpression of *AcMYB210* in kiwifruit ‘Donghong’. scale bar=1cm. **(E)** Detection of total anthocyanin content in WT and transgenic kiwifruit leaves. **(F)** Detection of the expression levels of *AcF3GT1a*, *AcF3GT4* and *AcGST1* in OE-AcMYB210 L1 and L2. Two-tailed Student’s*t*-test was conducted for statistical analysis and significant differences are marked with asterisks (**P < 0.01, ***P < 0.001).

Stable genetic transformation was conducted to further confirm the function of *AcMYB210* in kiwifruit. Obvious pigmentation was observed in OE-AcMYB210 Lines (L1 and L2) compared with WT (**Figure 6D**). Anthocyanin content in L1 and L2 lines were significantly higher than that in WT. In addition, the expression levels of *AcMYB210*, *AcF3GT1a*, *AcF3GT4*, and *AcGST1* in OE1 and OE2 were significantly higher than those in WT (**Figures 6E, F**). These results indicate that *AcMYB210* can directly regulate anthocyanin biosynthesis in kiwifruit leaves.

### 3.6 AcMYB210 interacts with AcbHLH5 to form an MBW complex

*AcMYB210* alone cannot induce anthocyanin biosynthesis in *N. tabacum* leaves (**Figure 6A**), but when co-expressed with *AcbHLH5*, it significantly promotes anthocyanin pigmentation. The Y2H assay was performed to verify the interaction between AcMYB210 and AcbHLH5 protein. The results showed that yeast cells co-transformed with AcMYB210-BD and AcbHLH5-AD grew well on SD/-Leu/-Trp/-His/-Ade plates, indicating that AcMYB210 interacts with AcbHLH5 (**Figure 7A**). This interaction was further confirmed by a luciferase complementation imaging (LCI) assay, in which co-infiltration of AcMYB210-NLUC and AcbHLH5-CLUC into *N. benthamiana* leaves produced a clear luminescent signal compared with the control (**Figure 7B**). However, there was no significant difference in the effects of AcMYB210 and AcbHLH5 on the promoters of *AcF3GT1a* and *AcGST1* compared to AcMYB210 alone (**Supplementary Figure S4**). This result is consistent with findings in peach PpMYB10.1, where expressing PpMYB10.1 alone into *N. tabacum* leaves fails to accumulate anthocyanins, but co-expression with the partner protein PpbHLH3 enables accumulation (Zhou et al., 2015). Our RNA-seq data showed that AcbHLH5 is expressed in kiwifruit leaves, which explains why over-expression of AcMYB210 alone is sufficient to promote anthocyanin accumulation in kiwifruit. Together, these results demonstrate that AcMYB210 and AcbHLH5 interact to form an MBW complex that promotes anthocyanin biosynthesis.

**Figure 7:**
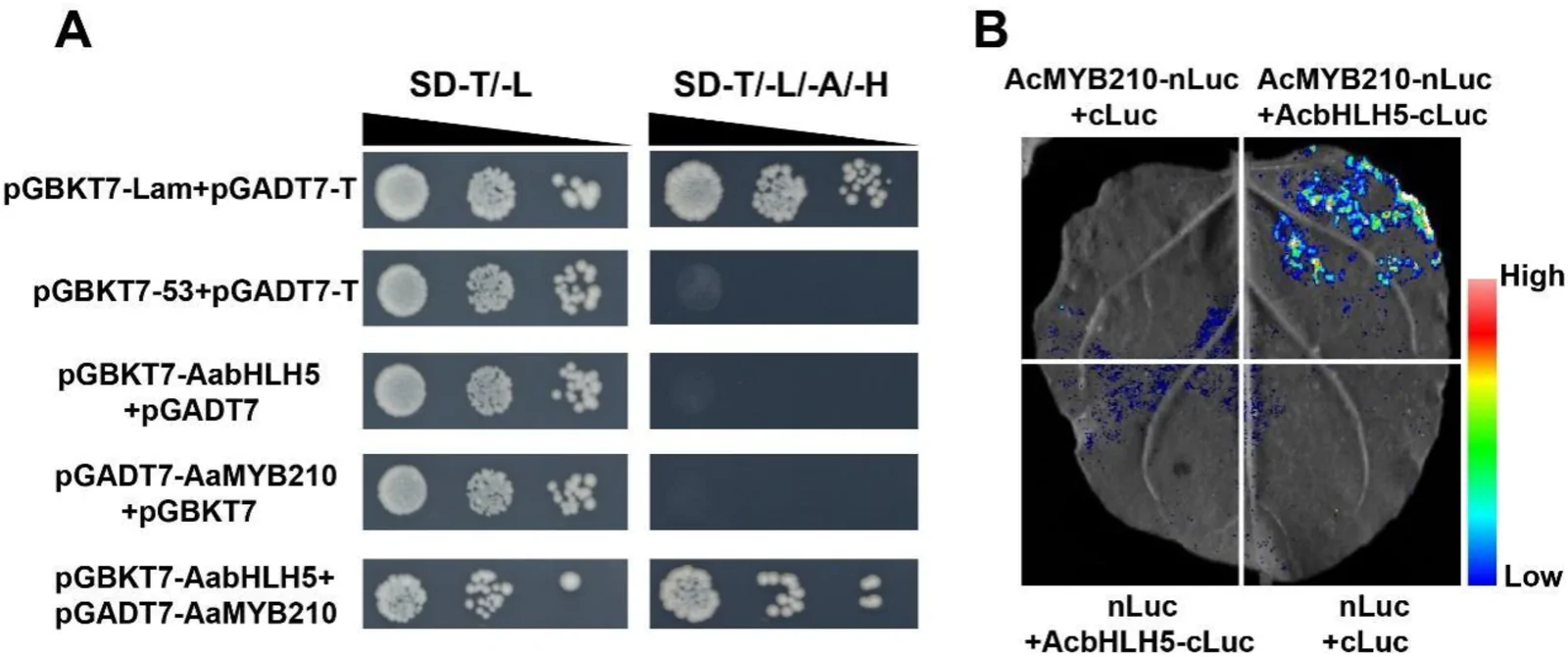
AcMYB210 interact with AcbHLH5. **(A)** Y2H assay showing the interaction between AcMYB210 and AcbHLH5. SD-T/-L represents for SD/-Trp/-Leu; SD-T/-L/-H/-A represents for SD-Trp/-Leu/-His/-Ade. **(B)** Firefly luciferase complementation imaging (LCI) in *N. benthamiana* leaves.

### 3.7 AcWRKY22 significantly activates the promoter activity of *AcMYB210*

To investigate the upstream regulatory factors that control *AcMYB210*, analysis was performed on the DEGs identified in the transcriptome, to find genes in the same expression pattern cluster as *AcMYB210*. The results showed that the DEGs were distributed across nine clusters, among which cluster 3 corresponded to the trends of both anthocyanin content and *AcMYB210* expression. AcMYB210 and ten other differentially expressed transcription factors (DETFs) (*AcNFYA1*, *AcbHLH093*, *AcCDF2*, *AcMYB4*, *AcERF12*, *AcMYB3*, *AcMYB56*, *AcGBF3*, *AcESE3*, *AcWRKY22*) were selected from cluster 3 based on criteria of FPKM ≥ 5 and log_2_FC ≥ 2. These ten DETFs were individually cloned into the pSAK277 vector, while the *AcMYB210* promoter was cloned into the dual-luciferase reporter vector (**Figure 8A**). It was found that *AcCDF2* and *AcMYB56* expression resulted in dead patches in *N. benthamiana* leaves. We therefore examined the regulatory effects of the remaining 8 DETFs on the *AcMYB210* promoter. The results showed that *AcWRKY22* could significantly activate the promoter activity of *AcMYB210*, with the LUC/REN ratio being approximately 9.1 times that of the EV. The Y1H was performed to further confirm whether AcWRKY22 binds to the promoter of *AcMYB210*, the results showed that on SD-Leu plates containing 200 ng/mL AbA, the strain containing AcWRKY22 grew normally, confirming that AcWRKY22 can interact with the promoter of *AcMYB210* (**Figure 8C**). These results suggest that AcWRKY22 responds to continuous light treatment and directly binds to and activates the promoter of *AcMYB210*.

**Figure 8:**
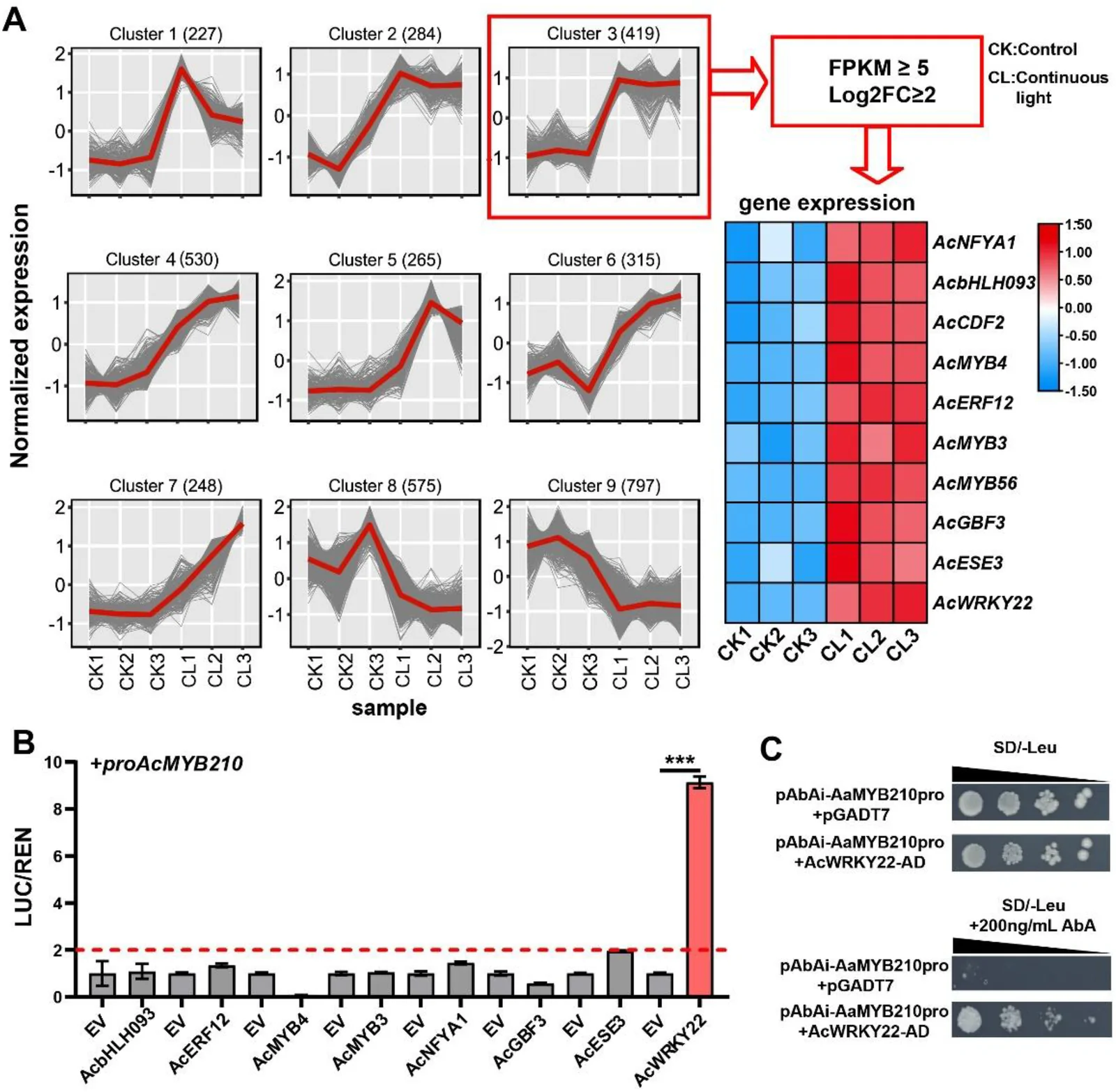
AcWRKY22 positively regulates the promoter of *AcMYB210*. **(A)** Trend analysis of DEGs from the transcriptome. **(B)** Effects of DETFs on the promoter activity of *AcMYB210 via* dual-luciferase assays. **(C)** Yeast one-hybrid (Y1H) assays confirmed the interaction of AcWRKY22 with the *AcMYB210* promoter. Two-tailed Student’s *t*-test was conducted for statistical analysis and significant differences are marked with asterisks (***P < 0.001).

### 3.8 *AcMYB210* also responds to waterlogging

Kiwifruit leaves show a reddening response to a number of other stresses. For instance, in kiwifruit plants recovering from waterlogging, some leaves exhibit a strong red coloration (**Figure 9A**). We analyzed the anthocyanin content (**Figure 9B**) and gene expression levels in these leaves and found that *AcMYB210* was significantly induced (**Figure 9C**). Although *AcMYB110* was also induced, its expression level was significantly lower than that of *AcMYB210* (**Figure 9C**). These results suggest that *AcMYB210* is likely a marker gene for anthocyanin accumulation in kiwifruit leaves under adverse growth conditions (**Figure 9D**).

**Figure 9:**
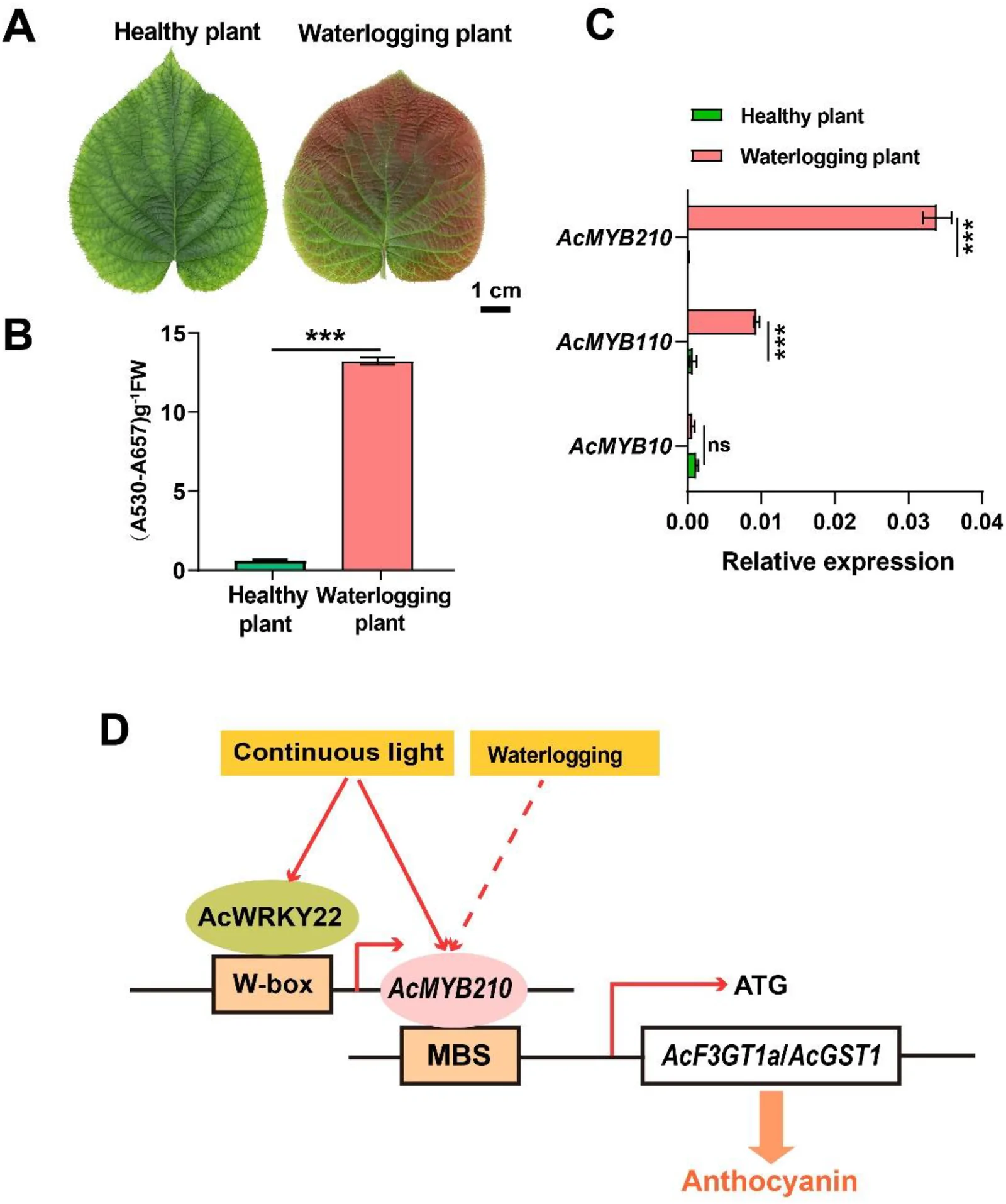
Anthocyanin accumulation in kiwifruit leaves after recovery from waterlogging. **(A)** Photographs of leaves from healthy plants and plants that experienced waterlogging. **(B)** Anthocyanin content in healthy plants and waterlogging plants. **(C)** Expression levels of *AcMYB210*, *AcMYB110*, and *AcMYB10* in healthy plants and waterlogged plants. **(D)** Schematic model of anthocyanin biosynthesis in kiwifruit leaves under continuous light and waterlogging conditions. Two-tailed Student’s *t*-test was conducted for statistical analysis and significant differences are marked with asterisks (***P < 0.001).

## 4 Discussion

### 4.1 AcF3GT1a, AcF3GT4 and AcGST1 may serve as key rate-limiting steps for anthocyanin synthesis in kiwifruit leaves.

Transcriptome profiling under continuous light treatment revealed that among the DEGs, two flavonoid 3-*O*-glucosyltransferases (*AcF3GT1a* and *AcF3GT4*) and one glutathione S-transferase (*AcGST1*) were strongly up-regulated. F3GTs catalyze the final glycosylation step that stabilizes anthocyanidins into anthocyanins, a reaction considered rate-limiting for anthocyanin accumulation in many species (Meng et al., 2019). In kiwifruit, the presence of multiple F3GT isoforms suggests functional redundancy, however our transcriptomic data indicated that *AcF3GT1a* and *AcF3GT4* are specifically induced by light, whereas other family members remain unchanged. Meanwhile, GSTs are responsible for transporting anthocyanins from the cytosolic site of synthesis into the vacuole (Cao et al., 2020), and loss-of-function mutations in GST genes result in pale or colorless phenotypes in petunia and *Arabidopsis* (Sun et al., 2012). *AcGST1* can be induced to express under light conditions, which indicates that AcGST1 is crucial for the synthesis of anthocyanins in the kiwifruit leaves. These results are consistent with our previous results in the fruit flesh of kiwifruit where AcF3GT1a and AcGST1 are limiting anthocyanin accumulation (Li et al., 2026). Other anthocyanin biosynthetic genes may have minor changes might be ignored due to sampling timing. While our current results supported that *AcF3GT1a*, *AcF3GT4*, and *AcGST1* are key genes controlling the light signaling pathway during elevation of anthocyanins in kiwifruit.

### 4.2 *AcMYB210* acts as the master R2R3-MYB regulator during light-dependent leaf reddening

R2R3 MYB transcription factors, particularly those of the S6 subfamily, are well-established as master switches of anthocyanin biosynthesis (Wu et al., 2022). Transcriptome data showed that among the three S6 MYB transcription factors (AcMYB10, AcMYB110, AcMYB210) belonging to the same subfamily as AtPAP1 in kiwifruit (Rodrigues et al., 2021), only *AcMYB210* showed significantly higher expression under continuous light treatment. Our Y1H, dual-luciferase assay collectively demonstrates that AcMYB210 directly binds to the promoters of *AcF3GT1a* and *AcGST1* and activates their transcription. Moreover, AcMYB210 possesses transcriptional activation activity and localizes exclusively to the nucleus when transiently expressed in tobacco epidermal cells, satisfying the criteria of a transcriptional activator. Transient overexpression in tobacco leaves led to obvious purple pigmentation, and stable transgenic kiwifruit lines overexpressing *AcMYB210* (we only got two lines as some callus are hard to re-generation when overexpression AcMYB210) showed markedly increased anthocyanin content compared with WT. These results establish AcMYB210 as a positive regulator of anthocyanin accumulation. This is analogous to the roles of *AtPAP1* in *Arabidopsis* (Guo et al., 2023), *MdMYB10* in apple (Zhang et al., 2023), and *PyMYB10* in pear (Feng et al., 2010), yet *AcMYB210* appears to be uniquely light-responsive in kiwifruit.

Phylogenetic analysis reveals that AcMYB210, together with AcMYB110, are tandem duplicates on linkage group 9, while AcMYB10 is their homeologue on linkage group 1 resulting from a recent whole-genome duplication specific to the kiwifruit lineage (Rodrigues et al., 2021). Functionally, SG6 MYB activators share conserved C-terminal motifs-designated S6A, S6B, and S6C-that exhibit molecular recognition feature (MoRF)-like characteristics and are critical for activation strength. However, functional variation in these motifs correlates with differential activation potency. AcMYB210, like AcMYB10 and AcMYB110, demonstrates strong anthocyanin-promoting activity (Rodrigues et al., 2021). Notably, while AcMYB210 and AcMYB110 are tandem duplicates, they may have undergone functional divergence (Pilkington et al., 2018), as suggested by their distinct expression patterns. This functional diversification within the kiwifruit SG6 clade underscores the evolutionary plasticity of anthocyanin regulatory networks and highlights the importance of investigating the specific functional roles of individual SG6 members in different developmental and environmental contexts.

### 4.3 AcMYB210 forms functional MBW regulatory complex *via* physical interaction with AcbHLH5

Our transient expression assays showed that tobacco leaves infiltrated with single AcMYB210 gene cannot produce visible pigmentation. Some SG6 MYB cannot accumulate anthocyanin in *N tabacum* leaves alone, such as peach *PpMYB10.1* and Chinese bayberry *MrMYB1*, it is similar with our identified AcMYB210. While co-infiltration of AcMYB210 and AcbHLH5 yielded markedly stronger anthocyanin accumulation, showing that AcMYB210 promotes anthocyanin synthesis by interacting with AcbHLH5 to form the MBW complex. The MBW complex constitutes the core transcriptional unit activating anthocyanin structural genes across plants, where direct protein-protein interaction between R2R3-MYB and bHLH partners dramatically amplifies target promoter transactivation efficiency (Xu et al., 2014, 2015). In the present study, Y2H assay and LCI in tobacco leaves independently validated a physical interaction between AcMYB210 and AcbHLH5. This is functionally analogous to AcMYB123-AcbHLH42 interaction that controls red flesh formation in ‘Hongyang’ kiwifruit (Wang et al., 2019), yet the AcMYB210 is a light-inducible activator in leaves, revealing tissue-specific MBW complex governed anthocyanin biosynthesis in different kiwifruit organs.

### 4.4 AcMYB210 is a marker gene for stress induced anthocyanin accumulation

Plants often accumulate anthocyanins under various stress conditions as an indicator of their physiological status (Jezek et al., 2023). Excessive light intensity and continuous light exposure are also forms of abiotic stress. Our results indicate that continuous light treatment induces anthocyanin accumulation in kiwifruit plants. In other species, S6 subfamily R2R3 MYB transcription factors have been reported to promote anthocyanin accumulation through regulation by light signaling pathway genes, such as when apple MdHY5 regulates MdMYB1 to promote anthocyanin synthesis (Fang et al., 2019b). Through clustal analysis of DEGs in the transcriptome, combined with dual-luciferase and yeast one-hybrid assays, *AcWRKY22* was identified as an upstream positive regulator of *AcMYB210*; however, the biological function of *AcWRKY22* in anthocyanin biosynthesis in kiwifruit remains to be further explored. HY5, as a master regulator of light signaling, has been shown to directly activate or repress the expression of WRKY transcription factors in multiple plant species; for example, AaHY5 activates *AaWRKY9* expression by binding to its promoter in *Artemisia annua* (Fu et al., 2021), and MdHY5 negatively regulates *MdWRKY41* expression in apple (Mao et al., 2021). Conversely, WRKY transcription factors can also regulate HY5; for instance, WRKY36 directly binds to the *HY5* promoter to inhibit its transcription (Yang et al., 2018), while MdWRKY11 binds to the MdHY5 promoter and promotes its activity (Liu et al., 2019). These findings suggest that *AcWRKY22* may similarly participate in light signaling networks, potentially through reciprocal regulation with HY5 or through integration with other light-responsive pathways, though direct experimental validation of this hypothesis in kiwifruit remains an important direction for future research. Based on the above findings, a linear regulatory hierarchy was established: light signaling - AcWRKY22 - AcMYB210/AcbHLH5 - AcF3GT/AcGST1. Furthermore, we found that *AcMYB210* also responds to waterlogging stress; however, the corresponding regulatory mechanism was not explored in this current study. Nevertheless, we conclude that AcMYB210 may serve as a marker gene for stress-induced anthocyanin accumulation.

## 5 Conclusion

Previous studies on anthocyanin biosynthesis in kiwifruit have primarily focused on fruit flesh. However, anthocyanin accumulation is frequently observed in kiwifruit leaves, and leaf pigmentation often reflects the overall health status of the plant. In this study, we induced anthocyanin accumulation in kiwifruit leaves through continuous light treatment and identified a previously uncharacterized MYB transcription factor, *AcMYB210*, which was significantly up-regulated under this treatment. Our results demonstrated that AcMYB210 interacts with AcbHLH5 to form a complex that promotes anthocyanin accumulation in kiwifruit leaves. Furthermore, we found that continuous light treatment activates the expression of *AcMYB210* through the transcription factor *AcWRKY22*, thereby transmitting the light signal. Additionally, *AcMYB210* was also induced in response to anthocyanin accumulation following waterlogging stress. Collectively, these findings suggest that *AcMYB210* may serve as a marker gene for stress-induced anthocyanin accumulation in kiwifruit, and this study establishes the AcWRKY22-AcMYB210 transcriptional cascade as a novel regulatory module governing light-responsive anthocyanin biosynthesis in kiwifruit.

## Data Availability

The data presented in the study are deposited in the National Genomics Date Center repository, accession number PRJCA074725.

## References

[B1] Alabd A. Ahmad M. Zhang X. Gao Y. Peng L. Zhang L. . (2022). Light-responsive transcription factor PpWRKY44 induces anthocyanin accumulation by regulating PpMYB10 expression in pear. Hortic. Res. 9, uhac199. doi: 10.1093/hr/uhac199 37180030 PMC10167416

[B2] Bai S. Tao R. Tang Y. Yin L. Ma Y. Ni J. . (2019). BBX16, a B-box protein, positively regulates light-induced anthocyanin accumulation by activating MYB10 in red pear. Plant Biotechnol. J. 17, 1985–1997. doi: 10.1111/pbi.13114 30963689 PMC6737026

[B3] Bursch K. Toledo-Ortiz G. Pireyre M. Lohr M. Braatz C. Johansson H. (2020). Identification of BBX proteins as rate-limiting cofactors of HY5. Nat. Plants 6, 921–928. doi: 10.1038/s41477-020-0725-0 32661279

[B4] Cao Y. Xu L. Xu H. Yang P. He G. Tang Y. . (2020). LhGST is an anthocyanin-related glutathione S-transferase gene in Asiatic hybrid lilies (*Lilium* spp.). Plant Cell Rep. 40, 85–95. doi: 10.1007/s00299-020-02615-y 33210154

[B5] Cappellini F. Marinelli A. Toccaceli M. Tonelli C. Petroni K. (2021). Anthocyanins: From mechanisms of regulation in plants to health benefits in foods. Front. Plant Sci. 12, 748049. doi: 10.3389/fpls.2021.748049 34777426 PMC8580863

[B6] Chen M. Chory J. (2011). Phytochrome signaling mechanisms and the control of plant development. Trends Cell Biol. 21, 664–671. doi: 10.1016/j.tcb.2011.07.002 21852137 PMC3205231

[B7] Fang H. Dong Y. Yue X. Chen X. He N. Hu J. . (2019a). MdCOL4 interaction mediates crosstalk between UV-B and high temperature to control fruit coloration in apple. Plant Cell Physiol. 60, 1055–1066. doi: 10.1093/pcp/pcz023 30715487

[B8] Fang H. Dong Y. Yue X. Hu J. Jiang S. Xu H. . (2019b). The B‐box zinc finger protein MdBBX20 integrates anthocyanin accumulation in response to ultraviolet radiation and low temperature. Plant Cell Environ. 42, 2090–2104. doi: 10.1111/pce.13552 30919454

[B9] Feng S. Wang Y. Yang S. Xu Y. Chen X. (2010). Anthocyanin biosynthesis in pears is regulated by a R2R3-MYB transcription factor PyMYB10. Planta 232, 245–255. doi: 10.1007/s00425-010-1170-5 20422209

[B10] Fu X. Peng B. Hassani D. Xie L. Liu H. Li Y. . (2021). AaWRKY9 contributes to light‐ and jasmonate‐mediated to regulate the biosynthesis of artemisinin in *Artemisia annua*. New Phytol. 231, 1858–1874. doi: 10.1111/nph.17453 33973259

[B11] Guo Y. Li D. Liu T. Li Y. Liu J. He M. . (2023). Genome-wide identification of PAP1 direct targets in regulating seed anthocyanin biosynthesis in *Arabidopsis*. Int. J. Mol. Sci. 24, 16049. doi: 10.3390/ijms242216049 38003239 PMC10671800

[B12] Hu D. G. Sun C. H. Ma Q. J. You C. X. Cheng L. Hao Y. J. (2016). MdMYB1 regulates anthocyanin and malate accumulation by directly facilitating their transport into vacuoles in Apples. Plant Physiol. 170, 1315–1330. doi: 10.1104/pp.15.01333 26637549 PMC4775115

[B13] Jezek M. Allan A. C. Jones J. J. Geilfus C. M. (2023). Why do plants blush when they are hungry? New Phytol. 239, 494–505. doi: 10.1111/nph.18833 36810736

[B14] Jiang W. Yin Q. Liu J. Su X. Han X. Li Q. . (2024). The APETALA2–MYBL2 module represses proanthocyanidin biosynthesis by affecting formation of the MBW complex in seeds of *Arabidopsis thaliana*. Plant Commun. 5, 100777. doi: 10.1016/j.xplc.2023.100777 38053331 PMC10943577

[B15] Kanehisa M. Sato Y. Kawashima M. Furumichi M. Tanabe M. (2016). KEGG as a reference resource for gene and protein annotation. Nucleic Acids Res. 44, D457–D462. doi: 10.1093/nar/gkv1070 26476454 PMC4702792

[B16] Kuang L. Chen J. Bao X. Zhang D. Liu J. Wang W. . (2025). Environmental and phytohormonal factors regulating anthocyanin biosynthesis in fruits. Horticulturae 11, 681. doi: 10.3390/horticulturae1106068130654563

[B17] Lee S.-H. Kim S.-H. Park T.-K. Kim Y.-P. Lee J.-W. Kim T.-W. (2024). Transcription factors BZR1 and PAP1 cooperate to promote anthocyanin biosynthesis in *Arabidopsis* shoots. Plant Cell 36, 3654–3673. doi: 10.1093/plcell/koae172 38869214 PMC11371145

[B18] Li B. Fan R. Guo S. Wang P. Zhu X. Fan Y. . (2019). The Arabidopsis MYB transcription factor, MYB111 modulates salt responses by regulating flavonoid biosynthesis. Environ. Exp. Bot. 166, 103807. doi: 10.1016/j.envexpbot.2019.10380742574925

[B19] Li X. Xu Q. Gulinuer A. Tian J. Zheng J. Chang G. . (2024). AcMYB96 promotes anthocyanin accumulation in onion (*Allium cepa* L) without forming the MBW complex. Plant Physiol. Biochem. 214, 108965. doi: 10.1016/j.plaphy.2024.108965 39067107

[B20] Li X. T. Huang Z. X. Zhai Y. X. Guo Y. T. Liu Y. T. Zhang Y. . (2026). An AcMYB10-AcF3GT1/GST1 module restricts anthocyanin synthesis in the fruit flesh of kiwifruit. Plant Cell Rep. 45, 131. doi: 10.1007/s00299-026-03828-3 42012690

[B21] Liu W. Wang Y. Yu L. Jiang H. Guo Z. Xu H. . (2019). MdWRKY11 participates in anthocyanin accumulation in red-fleshed apples by affecting MYB transcription factors and the photoresponse factor MdHY5. J. Agric. Food. Chem. 67, 8783–8793. doi: 10.1021/acs.jafc.9b02920 31310107

[B24] Liu Y. Tikunov Y. Schouten R. E. Marcelis L. F. M. Visser R. G. F. Bovy A. (2018). Anthocyanin biosynthesis and degradation mechanisms in solanaceous vegetables: A review. Front. Chem. 6. doi: 10.3389/fchem.2018.00052 29594099 PMC5855062

[B23] Liu Y. Ma K. Qi Y. Lv G. Ren X. Liu Z. . (2021). Transcriptional regulation of anthocyanin synthesis by MYB-bHLH-WDR complexes in Kiwifruit (*Actinidia chinensis*). J. Agric. Food. Chem. 69, 3677–3691. doi: 10.1021/acs.jafc.0c07037 33749265

[B22] Liu Y. Lv G. Yang Y. Ma K. Ren X. Li M. . (2023). Interaction of AcMADS68 with transcription factors regulates anthocyanin biosynthesis in red-fleshed kiwifruit. Hortic. Res. 10, uhac252. doi: 10.1093/hr/uhac252 36751270 PMC9896601

[B25] Ma Y. Ma X. Gao X. Wu W. Zhou B. (2021). Light induced regulation pathway of anthocyanin biosynthesis in plants. Int. J. Mol. Sci. 22, 11116. doi: 10.3390/ijms222011116 34681776 PMC8538450

[B26] Mao Z. Jiang H. Wang S. Wang Y. Yu L. Zou Q. . (2021). The MdHY5-MdWRKY41-MdMYB transcription factor cascade regulates the anthocyanin and proanthocyanidin biosynthesis in red-fleshed apple. Plant Sci. 306, 110848. doi: 10.1016/j.plantsci.2021.110848 33775373

[B27] Meng X. Li Y. Zhou T. Sun W. Shan X. Gao X. . (2019). Functional differentiation of duplicated flavonoid 3-O-Glycosyltransferases in the flavonol and anthocyanin biosynthesis of *Freesia hybrida*. Front. Plant Sci. 10. doi: 10.3389/fpls.2019.01330 31681396 PMC6813240

[B28] Mi H. Muruganujan A. Ebert D. Huang X. Thomas P. D. (2019). PANTHER version 14: more genomes, a new PANTHER GO-slim and improvements in enrichment analysis tools. Nucleic Acids Res. 47, D419–D426. doi: 10.1093/nar/gky1038 30407594 PMC6323939

[B29] Naing A. H. Kim C. K. (2021). Abiotic stress‐induced anthocyanins in plants: Their role in tolerance to abiotic stresses. Physiol. Plant 172, 1711–1723. doi: 10.1111/ppl.13373 33605458

[B30] Pilkington S. M. Crowhurst R. Hilario E. Nardozza S. Fraser L. Peng Y. . (2018). A manually annotated *Actinidia chinensis* var. *chinensis* (kiwifruit) genome highlights the challenges associated with draft genomes and gene prediction in plants. BMC Genomics 19, 257. doi: 10.1186/s12864-018-4656-3 29661190 PMC5902842

[B31] Rodrigues J. A. Espley R. V. Allan A. C. (2021). Genomic analysis uncovers functional variation in the C-terminus of anthocyanin-activating MYB transcription factors. Hortic. Res. 8, 77. doi: 10.1038/s41438-021-00514-1 33790254 PMC8012628

[B32] Song Z. Li Y. Zhan X. Li X. Ye L. Lin M. . (2025). AaMYB61-like and AabHLH137 jointly regulate anthocyanin biosynthesis in *Actinidia arguta*. BMC Plant Biol. 25, 89. doi: 10.1186/s12870-025-06109-5 39844047 PMC11753137

[B33] Sun Y. Li H. Huang J.-R. (2012). Arabidopsis TT19 functions as a carrier to transport anthocyanin from the cytosol to tonoplasts. Mol. Plant 5, 387–400. doi: 10.1093/mp/ssr110 22201047

[B35] Wang W. Q. Moss S. M. A. Zeng L. Espley R. V. Wang T. Lin‐Wang K. . (2022). The red flesh of kiwifruit is differentially controlled by specific activation–repression systems. New Phytol. 235, 630–645. doi: 10.1111/nph.18122 35348217

[B34] Wang L. Tang W. Hu Y. Zhang Y. Sun J. Guo X. . (2019). A MYB/bHLH complex regulates tissue‐specific anthocyanin biosynthesis in the inner pericarp of red‐centered kiwifruit *Actinidia chinensis* cv. Hongyang. Plant J. 99, 359–378. doi: 10.1111/tpj.14330 30912865

[B36] Wu Y. Wen J. Xia Y. Zhang L. Du H. (2022). Evolution and functional diversification of R2R3-MYB transcription factors in plants. Hortic. Res. 9, uhac058. doi: 10.1093/hr/uhac058 35591925 PMC9113232

[B37] Xing M. Xin P. Wang Y. Han C. Lei C. Huang W. . (2024). A negative feedback regulatory module comprising R3-MYB repressor MYBL2 and R2R3-MYB activator PAP1 fine-tunes high light-induced anthocyanin biosynthesis in *Arabidopsis*. J. Exp. Bot. 75, 7381–7400. doi: 10.1093/jxb/erae399 39303008

[B38] Xu W. Dubos C. Lepiniec L. (2015). Transcriptional control of flavonoid biosynthesis by MYB-bHLH-WDR complexes. Trends Plant Sci. 20, 176–185. doi: 10.1016/j.tplants.2014.12.001 25577424

[B39] Xu W. Grain D. Bobet S. Le Gourrierec J. Thévenin J. Kelemen Z. . (2014). Complexity and robustness of the flavonoid transcriptional regulatory network revealed by comprehensive analyses of MYB-bHLH-WDR complexes and their targets in *Arabidopsis* seed. New Phytol. 202, 132–144. doi: 10.1111/nph.12620 24299194

[B40] Yang Y. Liang T. Zhang L. Shao K. Gu X. Shang R. . (2018). UVR8 interacts with WRKY36 to regulate HY5 transcription and hypocotyl elongation in *Arabidopsis*. Nat. Plants 4, 98–107. doi: 10.1038/s41477-017-0099-0 29379156

[B41] Yin R. Ulm R. (2017). How plants cope with UV-B: from perception to response. Curr. Opin. Plant Biol. 37, 42–48. doi: 10.1016/j.pbi.2017.03.013 28411583

[B42] Yu M. Xiong J. Dong K. Quan X. Guo H. Huo J. . (2024). AcMYB10 involved in anthocyanin regulation of ‘Hongyang’ kiwifruit induced via fruit bagging and high-postharvest-temperature treatments. Genes 15, 97. doi: 10.3390/genes15010097 38254986 PMC10815172

[B43] Zhang S. Jiao Z. Liu Z. Peng Z. Cui L. Hu Z. . (2026). Multiomics analysis revealed the role of LsBBX22–LsHY5a/b module in promoting UV–B-mediated anthocyanin accumulation in Lettuce. J. Agric. Food. Chem. 74, 1788–1803. doi: 10.1021/acs.jafc.5c08530 41480951

[B44] Zhang S. Wang H. Wang T. Liu W. Zhang J. Fang H. . (2023). MdMYB305-MdbHLH33-MdMYB10 regulates sugar and anthocyanin balance in red-fleshed apple fruits. Plant J. 113, 1062–1079. doi: 10.1111/tpj.16100 36606413

[B45] Zheng X.-T. Yu Z.-C. Tang J.-W. Cai M.-L. Chen Y.-L. Yang C.-W. . (2020). The major photoprotective role of anthocyanins in leaves of *Arabidopsis thaliana* under long-term high light treatment: antioxidant or light attenuator? Photosynth. Res. 149, 25–40. doi: 10.1007/s11120-020-00761-8 32462454

[B46] Zhou H. Lin-Wang K. Wang H. Gu C. Dare A. P. Espley R. V. . (2015). Molecular genetics of blood-fleshed peach reveals activation of anthocyanin biosynthesis by NAC transcription factors. Plant J. 82, 105–121. doi: 10.1111/tpj.12792 25688923

[B47] Zhou L.-L. Shi M.-Z. Xie D.-Y. (2012). Regulation of anthocyanin biosynthesis by nitrogen in TTG1–GL3/TT8–PAP1-programmed red cells of *Arabidopsis thaliana*. Planta 236, 825–837. doi: 10.1007/s00425-012-1674-2 22669605

