# Supplementary material for: AcWRKY22 activates AcMYB210 to regulate light induced anthocyanin accumulation in kiwifruit leaves
Source: Front Plant Sci. 2026 Oct 5;17:1944909. doi: 10.3389/fpls.2026.1944909 (PMC13677498; doi:10.3389/fpls.2026.1944909)
Supplement: Supplementary file 1 [file SupplementaryFile2.docx]

Primers for qRT-PCR:

| No. | Gene name | Forward primers (5’ to 3’) | Reverse primers (5’ to 3’) |
| --- | --- | --- | --- |
| 1 | Nz-Actin | CCAAGGCCAACAGAGAGAAG | GACGGAGGATAGCATGAGGA |
| 2 | Acc20131 | CTGAAGGTAGCGGCAAAGGG | ATCCAAATTGGCACCCACGA |
| 3 | Acc20132 | TGCAAGCGGTTGAAGTAGAGA | CCAAATTGGCACCCACGAGA |
| 4 | Acc10479 | AGGTAGAGGCGCACAACTTC | GAGTGCCAAGTCACTACGCT |
| 5 | Acc10232 | GAGCCGATACCCAAAACCCA | GGGATGGCTTGGGGAGGTTA |
| 6 | Acc00493 | AAGGTACAGAGGCCCAACCT | TCACAGTCTTGTTGTCCCACC |
| 7 | Acc10227 | GGTCAACCGCCTGCTGAATA | AGCTCGAGGGTCCCCATTT |
| 8 | Nt-Actin | AATGGAACTGGAATGGTCAAGGC | TGCCAGATCTTCTCCATGTCATCCCA |
| 9 | NtCHS | TTGTTCGAGCTTGTCTCTGC | AGCCCAGGAACATCTTTGAG |
| 10 | NtCHI | GTCAGGCCATTGAAAAGCTC | CTAATCGTCAATGCCCCAAC |
| 11 | NtF3H | CAAGGCATGTGTGGATATGG | TGTGTCGTTTCAGTCCAAGG |
| 12 | NtF3'H | AGGCTCAACACTTCTCGT | CATCAACTTTGGGCTTCT |
| 13 | NtDFR1 | AACCAACAGTCAGGGGAATG | TTGGACATCGACAGTTCCAG |
| 14 | NtANS | TGGCGTTGAAGCTCATACTG | GGAATTAGGCACACACTTTGC |
| 15 | NtUFGT | GAGTGCATTGGATGCCTTTT | CCAGCTCCATTAGGTCCTTG |

Primers for vector construction:

| *No.* | *Vector* | *Gene* | *Primer Sequence 5'-3'* |
| --- | --- | --- | --- |
| 1 | LUC | Acc20131-F | tcgacggtatcgataagcttgaccgacttagatcaacaactgatcttt |
| 2 | LUC | Acc20131-R | tgtttttggcgtcttccatAGGCTTTGATTTTGGGTCTAGCTTGTGG |
| 3 | LUC | Acc20132-F | tcgacggtatcgataagcttttaacttgtttctaaattaaactattct |
| 4 | LUC | Acc20132-R | tgtttttggcgtcttccatGGTTTTTTAAGGCTTTGATTTTGGGTCT |
| 5 | LUC | Acc10479-F | tcgacggtatcgataagcttagatgctaattagttaacacattaaat |
| 6 | LUC | Acc10479-R | tgtttttggcgtcttccatACCCTAGCAAGTGCTCTTAAATAGAAA |
| 7 | Psak277 | Acc11714-F | ggatccaaagaattcATGGAGTATACTCATGAACATGGT |
| 8 | Psak277 | Acc11714-R | agaagtactctcgagCTACAAGCACCTTCCTCCATATCC |
| 9 | Psak277 | Acc23044-F | ggatccaaagaattcATGAACGGTGGAGATGCGTTGGAG |
| 10 | Psak277 | Acc23044-R | agaagtactctcgagTTATGTGCTCTCTTGGAACGTATG |
| 11 | Psak277 | Acc18964-F | ggatccaaagaattcATGGAGACGCATTACAGAGGAGTG |
| 12 | Psak277 | Acc18964-R | agaagtactctcgagCTACATATCTCGCAGCGGGTAAAT |
| 13 | Psak277 | Acc14071-F | ggatccaaagaattcATGAGGAAGTCATGCTGTGAGAAG |
| 14 | Psak277 | Acc14071-R | agaagtactctcgagTCACATAAAGAGAAGTAGGGTAGG |
| 15 | Psak277 | Acc26582-F | ggatccaaagaattcATGAAGAAGATGACGACGACAA |
| 16 | Psak277 | Acc26582-R | agaagtactctcgagTCACTGCAGTTGATATAGCTTC |
| 17 | Psak277 | Acc13155-F | ggatccaaagaattcATGCATCAAAGATCGGAAAGCACA |
| 18 | Psak277 | Acc13155-R | agaagtactctcgagTTATTCGGAATAAAATGATGACTT |
| 19 | Psak277 | Acc23046-F | ggatccaaagaattcATGAGTCCACAACAGTCCAACTTC |
| 20 | Psak277 | Acc23046-R | agaagtactctcgagTTAAGCCGCTCCTACTCCAAGGAA |
| 21 | Psak277 | Acc15944-F | ggatccaaagaattcATGGGTAATAGTGAGGAGACAA |
| 22 | Psak277 | Acc15944-R | agaagtactctcgagTCATCCCGCAGCTAAGACATCC |
| 23 | Psak277 | Acc29209-F | ggatccaaagaattcATGGCCAGACCACAACAGCGATAC |
| 24 | Psak277 | Acc29209-R | agaagtactctcgagTCACATATCCTGAGAAGGCACAAC |
| 25 | 62SKBD | Acc10227-F | CGCCGTCTAGAACTAGTGGATCCATGGAAAGTGTTCTTGTAGGAAT |
| 26 | 62SKBD | Acc10227-R | TCGATAAGCTTGATATCGAATTCTTACTGCTTGTCCATACCTAAAA |
| 27 | pbinGFP | Acc10227-F | TACAAGGGTACCCCCATGGAAAGTGTTCTTGTAGGAAT |
| 28 | pbinGFP | Acc10227-R | GGATCCGTCGACCCCTTACTGCTTGTCCATACCTAAAA |
| 29 | pGBKT7 | Acc10227-F | ATGGCCATGGAGGCCGAATTCATGGAAAGTGTTCTTGTAG |
| 30 | pGBKT7 | Acc10227-R | CCGCTGCAGGTCGACCTGCTTGTCCATACCTAAAA |
| 31 | pGADT7 | Acc19563-F | GCCATGGAGGCCAGTGAATTCATGGCGGCTCCCCCTAGTAC |
| 32 | pGADT7 | Acc19563-R | CAGCTCGAGCTCGATGGATCCGGGTATGATTTGGTGTATTGCTC |
| 33 | nLUC | Acc10227-F | CTCGGTACCCGGGGATCCATGGAAAGTGTTCTTGTAG |
| 34 | nLUC | Acc10227-R | GTACGAGATCTGGTCGACCTGCTTGTCCATACCTAAA |
| 35 | cLUC | Acc19563-F | GCGTCCCGGGGCGGTACCATGGCGGCTCCCCCTAGTAC |
| 36 | cLUC | Acc19563-R | AGTCCATTTGTTGGATCCGGGTATGATTTGGTGTATTGCTC |
| 37 | pGADT7 | Acc10227-F | GCCATGGAGGCCAGTGAATTCATGGAAAGTGTTCTTGTAG |
| 38 | pGADT7 | Acc10227-R | CAGCTCGAGCTCGATGGATCCCTGCTTGTCCATACCTAAA |
| 39 | pAbAi | Acc10227-F | cttgaattcgagctcggtaccTTTAGATATGTATTTTTCGT |
| 40 | pAbAi | Acc10227-R | atacagagcacatgcctcgagATCCGACACTACACTTGAGTA |
| 41 | pAbAi | Acc20131-F | cttgaattcgagctcggtaccGACCGACTTAGATCAACAACT |
| 42 | pAbAi | Acc20131-R | atacagagcacatgcctcgagTTTTGGGTCTAGCTTGTGGT |
| 43 | pAbAi | Acc10479-F | cttgaattcgagctcggtaccAGATGCTAATTAGTTAACACA |
| 44 | pAbAi | Acc10479-R | atacagagcacatgcctcgagACCCTAGCAAGTGCTCTTAAA |
