# Supplementary material for: AcWRKY22 activates AcMYB210 to regulate light induced anthocyanin accumulation in kiwifruit leaves
Source: Front Plant Sci. 2026 Oct 5;17:1944909. doi: 10.3389/fpls.2026.1944909 (PMC13677498; doi:10.3389/fpls.2026.1944909)
Supplement: Supplementary file 2 [file SupplementaryFile1.doc]

Figure S1. Statistical analysis of differentially expressed genes in the transcriptome.

Figure S2. Clustering analysis of differentially expressed genes

Figure S4. The expression of *AcbHLH5* in kiwifruit leaf under continuous light treatment

Figure S4. Regulatory effect of *AcMYB210* and AcbHLH5 on the promoters of *AcF3GT1a*, and *AcGST1*. Two-tailed Student’s *t*-test was conducted for statistical analysis and significant differences are marked with asterisks (ns: no significant).
